# Beyond social disorganization: Family SES, neighborhood context, and adolescent alcohol use

**DOI:** 10.1016/j.ssmph.2026.101960

**Published:** 2026-08-18

**Authors:** Albert J. Ksinan, Daniel Szabó, Lenka Andrýsková, Martin Bobak, Hynek Pikhart

**Affiliations:** a RECETOX, Faculty of Science, Masaryk University, Brno, Czech Republic; b Institute of Epidemiology & Health Care, University College London, London, United Kingdom

## Abstract

The relationship between contextual factors, including neighborhood characteristics, and adolescent alcohol use remains unclear, with studies finding either no effect or associations in either direction. The current study used data from a subset of a Czech longitudinal cohort (ELSPAC; analytic N = 1528) to examine how family socioeconomic status (SES), neighborhood advantage (administrative data), and perceived neighborhood disorder (subjective ratings) jointly influence alcohol use at age 15 (latent factor using three reports as indicators). Parental drinking, maternal monitoring, and peer alcohol use were considered as proximal mediators.

The results from structural equation modeling showed that, contrary to social disorganization theory, perceived neighborhood disorder was associated with lower adolescent alcohol use (β = −0.10, *p* = .007), while neighborhood advantage was not significantly associated with either alcohol use or the proximal mediators. Family SES showed no direct association with alcohol use but was indirectly associated with more drinking through lower perceived neighborhood disorder, greater parental alcohol use, and greater maternal monitoring (total indirect β = 0.017, 95% CI [0.004, 0.036]). Peer alcohol use remained the strongest correlate of adolescent drinking (β = 0.33, *p* < .001).

These findings are inconsistent with the social disorganization theory but are in line with many existing studies. Both higher SES and greater perceived neighborhood disorder predicted increased maternal monitoring, indicating two distinct pathways to supervision: one driven by resources, the other by environmental risk. These results highlight the value of modeling subjective neighborhood perceptions alongside family-level mediators for understanding adolescent alcohol use.

## Highlights

• Family SES predicted adolescent drinking via parental alcohol use and monitoring.
• Perceived neighborhood disorder was linked to lower adolescent alcohol use.
• Parental monitoring was affected by family SES and perceived neighborhood risk.
• Peer alcohol use strongly predicts adolescent drinking, independent of neighborhood.
• No effects found for objectively assessed neighborhood advantage.

## 1 Introduction

Adolescence is a critical developmental period during which most individuals initiate alcohol use, and an earlier onset has been consistently linked to an increased risk of later alcohol-related problems, including alcohol use disorder (Nguyen-Louie et al., 2024; Yuen et al., 2020). Adolescent alcohol use is a behavior stemming from multiple factors operating across different ecological contexts. Socioecological models emphasize that individual or family risk factors do not fully account for adolescent drinking without considering the effects of the physical and social environment they live in.

According to Bronfenbrenner, socialization occurs within nested systems, including the family, peer group, or neighborhood (Bronfenbrenner, 1977). Among these, family socioeconomic status (SES) and neighborhood characteristics have received increasing attention as important contextual influences on adolescent health behaviors. Family SES may shape the environments in which adolescents grow up, which in turn can affect parenting practices, peer relationships, and opportunities for risky behavior. However, findings on neighborhood effects remain mixed, in part due to inconsistent measurement approaches and limited integration of both objective and subjective indicators. To effectively prevent or delay alcohol use during adolescence, it is essential to model these various influences simultaneously in order to obtain a more comprehensive understanding of how contextual factors jointly shape adolescent alcohol use.

### 1.1 SES and alcohol use

Socioeconomic status (SES) reflects an individual's or family's social and economic position relative to other members of society, typically based on income, attained education, and occupational status. Higher levels of SES have been consistently found to be positively associated with higher levels of alcohol drinking (Collins, 2016; B. A. Martin et al., 2009; C. C. Martin, 2019; Patrick et al., 2012). This association has been mostly explained by access, as higher SES families have more disposable income to spend on alcohol (B. A. Martin et al., 2009), and more permissive alcohol-related norms (Pedersen et al., 2018). However, this association diverges for different alcohol-related outcomes. Although higher SES is associated with more alcohol drinking (both consumption and frequency), binge drinking is more prevalent among individuals with lower SES (Allen et al., 2018), and alcohol-attributable mortality is also more prevalent in this group (Probst et al., 2020).

### 1.2 SES and neighborhood characteristics

The characteristics of neighborhoods are affected by the individual characteristics of their inhabitants, often being simply aggregations of the characteristics of individual members. In this way, neighborhoods considered advantageous are usually characterized by significant proportion of families with high SES – and vice versa. There is most likely a self-reinforcing cycle in which individuals with high SES preferentially select into advantageous neighborhoods, and the resources available within such neighborhoods subsequently help reproduce that same status over time (Ksinan & Vazsonyi, 2021; Sampson & Sharkey, 2008; Savolainen et al., 2017).

The most studied neighborhood characteristic is neighborhood disadvantage, characterized by high rates of poverty, unemployment, single parenthood, and low levels of attained education (Ross & Mirowsky, 2001). Usually, objective, administrative measures (such as census-based indicators) of neighborhood are used to operationalize (dis)advantage of a particular neighborhood (e.g., unemployment rate, percentage of college-educated adults). However, there are also measures based on individual reports that capture how residents perceive their surroundings. These perceptual measures often reflect immediate social processes, safety, and physical disorder that objective data may miss (Harlow et al., 2025), leading some authors to argue that these subjective perceptions might be more important for capturing the effects of neighborhoods on problem behaviors than administrative measures (Martin-Storey et al., 2013; Zuberi, 2016). An example of a subjectively assessed neighborhood characteristic is neighborhood disorder, a resident's perception of own neighborhood with regards to social (vandalism, petty crime) and physical disorganization (litter, noise etc.; Ross & Mirowsky, 1999). Neighborhood disorder is closely related to neighborhood disadvantage, as neighborhoods with lack of economic and social resources are more likely to show signs of physical decay and social disarray, which generates noxious and stressful environment (Latkin & Curry, 2003).

### 1.3 Neighborhood characteristics and alcohol use

Social disorganization theory proposes that individuals living in disadvantaged and disordered neighborhoods would be more likely to engage in problem behaviors (Shaw & McKay, 1942). For example, Jackson et al. (2016) argued that concentrated neighborhood disadvantage may erode residents’ collective efficacy (shared norms and supervision important for inhibiting deviant behavior among young people), thereby increasing adolescent risk behaviors, including alcohol use. There have been many studies that have put this theory to test, with mixed findings regarding the effect of neighborhood effects on alcohol consumption (Jackson et al., 2014; Karriker-Jaffe, 2011). Several findings are in line with the Social disorganization theory (Bernburg et al., 2009; Fairman et al., 2020; Jackson et al., 2016), some studies found null effects (e.g., Brenner et al., 2011; Åslund & Nilsson, 2013), and a substantial amount of studies found a positive association between neighborhood advantage and alcohol use, mirroring the association found for individual SES. For example, the study by Slutske et al. (2016) found that neighborhoods with greater socioeconomic advantage showed higher levels of alcohol consumption. A study from Norway found that more affluent city districts have been associated with higher levels of alcohol use among adolescents (Pedersen et al., 2015). Similarly, other studies have fond higher levels of alcohol use in higher SES neighborhoods (Trim & Chassin, 2008), or lower levels of alcohol use in disadvantaged neighborhoods (Demidenko et al., 2024).

Comparatively fewer studies focused on the effects of neighborhood disorder. There have been several studies that found no effect of community disorganization/disorder (Byrnes et al., 2007; Fagan et al., 2007) and some other studies that did find positive association with substance use. Greater perception of neighborhood disorder was associated with greater perceived ease of purchasing cannabis, tobacco, and alcohol, as well as greater odds of using these substances (Harlow et al., 2025). Jackson et al. (2016) found that neighborhood physical disorder mediated the link between neighborhood disadvantage and binge drinking. In a longitudinal study, Barr (2018) found that although individuals from more disordered neighborhoods were at greater initial risk for higher levels of alcohol use, they showed lower levels of growth during from adolescence to young adulthood compared to individuals from lower disorder neighborhoods.

### 1.4 Proximal factors for alcohol use

The neighborhood effects on alcohol use in adolescence are hypothesized to emerge through more proximal factors (Jackson et al., 2014). Social ecological and developmental models emphasize that parents and peers are the immediate contexts of adolescent behavior and as such serve as intervening factors between neighborhood effects and individual alcohol use (Booth & Shaw, 2023). Among the most salient proximal ones identified in literature are parental alcohol use, parental monitoring, and peer alcohol use.

#### 1.4.1 Parental alcohol use

Parental alcohol use has been consistently found to positively affect adolescent alcohol use. This effect is partly genetic (Dick & Bierut, 2006; Liu et al., 2019) and partly through modeling behavior and permissive norms and attitudes towards alcohol (Abar et al., 2009; Ksinan et al., 2023; Latendresse et al., 2008). Regarding the effect between neighborhood and parental drinking, it has been found that neighborhoods that are more advantageous showed higher levels of alcohol drinking not just among the adolescents, but among their parents as well (Trim & Chassin, 2008), confirming the already existing positive link between family SES and alcohol use frequency.

#### 1.4.2 Parental monitoring

Parental monitoring has been repeatedly found to be one of the most important parenting practices, as higher levels of monitoring are protective factor for adolescent problem behaviors (DiClemente et al., 2001; Dishion & McMahon, 1998), including alcohol use. Previous studies have found that neighborhood disadvantage affected parenting behaviors (Leventhal & Brooks-Gunn, 2000; Sampson et al., 2002), yet it is unclear whether the association between parental monitoring and neighborhood disadvantage is positive, where living in disadvantaged neighborhood triggers more restrictive parental strategies (Zuberi, 2016) or negative, as found in recent study where neighborhood disadvantage was found to decrease the effectiveness of parental monitoring through perceived lower levels of neighborhood cohesion (Booth & Shaw, 2023).

#### 1.4.3 Peer alcohol use

The characteristics of adolescent's peer group have strong effects on their own behavior, especially related to risk behaviors. Specifically, affiliating with alcohol drinking peers predicts higher rates of alcohol use (Cruz et al., 2012; Liu et al., 2023). Neighborhoods that are disadvantaged often lack spaces and facilities where adolescents can spend their free time (such as recreational facilities), and this unstructured time has been associated with higher risk for substance use (Sznitman & Engel-Yeger, 2017). Furthermore, neighborhoods perceived as disorganized make it easier for adolescents to obtain alcohol, as they usually lack the informal social control for preventing and persecuting outlets and individuals offering alcohol to underage adolescents (Morrison et al., 2016; Romley et al., 2007).

## 2 Current study

The existing studies on neighborhood effects on adolescent alcohol use have produced mixed findings, with a substantial proportion of null findings. One limitation of this literature is that neighborhood and family socioeconomic status are often modeled as parallel, competing domains rather than as part of an explicit developmental pathway. As noted above, families with higher SES tend to select into more advantaged neighborhoods, so family and neighborhood SES are closely related, with neighborhood SES shaped in part by family SES.

Second, how a neighborhood is perceived by its residents might be more salient for adolescent outcomes than objective indicators alone, and relatively few studies have incorporated both subjective and objective neighborhood measures within the same model to examine their distinct or complementary contributions. Finally, the main effects of neighborhood characteristics on alcohol use are usually modest. Their effects might be more salient as part of a downstream process where they affect more proximal processes and contexts to adolescents (Leventhal & Brooks-Gunn, 2000).

The present study seeks to overcome the existing limitations by investigating the pathways by which socioeconomic status (SES) influences adolescent alcohol use via multiple neighborhood characteristics, measured both objectively and subjectively. These neighborhood characteristics are expected to shape parenting behaviors—specifically, parental alcohol use and maternal monitoring—as well as adolescents’ involvement with peers using alcohol. By testing this integrative model, the study aims to clarify the relative contributions of multiple environments, including family, peer, and neighborhood influences in the etiology of adolescent alcohol use.

Therefore, this study adopts a comprehensive framework with baseline family SES as a distal factor, neighborhood conditions (both objective neighborhood advantage and subjective neighborhood disorder), proximal mediators of their effects, and alcohol use at age 15. We hypothesize that neighborhood advantage and disorder affect adolescent drinking indirectly via family and peer processes, representing the proximal mediators of the contextual effects. For family processes, we hypothesize that maternal and paternal alcohol use would serve as risk factors while maternal monitoring would serve as a protective factor for adolescent drinking. For peer processes, we hypothesize that higher involvement with peers who drink alcohol would be associated with higher levels of adolescent drinking. This ordering (SES → neighborhood → proximal mediators → alcohol use) is consistent with the socioecological theory and existing evidence, and it allows us to test the mediational pathways by which socioeconomic and contextual factors shape adolescent alcohol behavior.

Finally, an important contribution of this study is testing this integrative model in the Czech Republic, a country characterized by high rates of underage drinking and permissive societal attitudes toward adolescent alcohol use (Hodačová et al., 2017; Ksinan et al., 2023), thereby extending existing neighborhood-effects research beyond its predominantly Western origins. Although much less work has been done on neighborhood effects within the Czech context, there are some studies suggesting that perceived neighborhood characteristics were associated with higher rates of alcohol misuse among adolescents (Spilkova et al., 2014), while another study of the general Czech population found area-level divorce rate, but not other area-level socioeconomic indicators, to be associated with alcohol use (Dzúrová et al., 2010).

## 3 Data and methods

### 3.1 Sample

Data for the present study come from the Czech arm of the European Longitudinal Study of Pregnancy and Childhood (ELSPAC), a multinational WHO-initiated cohort study. The baseline data included data from 5151 mothers and their newborns from the towns of Brno and Znojmo followed longitudinally starting in 1991 until children were 19 years of age. Throughout the study, the parents provided responses to questionnaires including various topics related to health, lifestyle, family, psychosocial factors, or environmental exposures. Beyond parental questionnaires, the data also includes reports from pediatricians, teachers, and starting at age 11, from children themselves (Piler et al., 2017). For this study, we focused on the timepoint when adolescents were 15 years old (N = 1691 due to sample attrition – see Piler et al., 2017). Limiting the sample to individuals who had their current address present resulted in an analytic sample of N = 1528 individuals (see Plan of analysis for missing data handling description). The sample was predominantly Czech (more than 99% of parents’ background) and included only participants from Brno, the second-largest city in the Czech Republic, with approximately 400,000 inhabitants, located in the South Moravian region. The city is characterized by a large student population and a growing technology sector, with strengths in software, semiconductors, and biotechnology. It shows substantial heterogeneity in neighborhood quality and affordability, ranging from socially excluded areas to affluent neighborhoods, with housing demand and prices driven up in recent years by an influx of new residents (Emmer et al., 2026; Hübelová et al., 2026).

The subsample was not statistically significantly different from the baseline sample in terms of sex ratio (χ2(1) = 2.48, *p* = .115) or family structure (χ2(1) < 0.01, *p* = 1). Both mothers and fathers had higher attained education in the subsample compared to the baseline (both *p* < .001), and the father's occupation status (indexed by ISEI) was higher in the subsample (*t* (1397.8) = 9.78, *p* < .001). Informed consent was obtained from all participants in the study. The secondary use of all ELSPAC study data was approved by the (C)ELSPAC Ethics Committee at Masaryk University in Brno, Czech Republic (Ref. No. ELSPAC/EK/1/2014, date 09/17/2014).

### 3.2 Measures

#### 3.2.1 Outcome

*Adolescent alcohol use.* Adolescent alcohol use at age 15 was operationalized as a latent factor indicated by reports from three sources: adolescent, mother, and pediatrician. Adolescents answered the question *How many times have you used the following drug: alcohol*, with options 1 = I use frequently, 2 = More than once in lifetime, 3 = once in lifetime, 4 = never. Mothers answered the question *Do you think your child drinks alcohol?* with options 1 = no, he/she despises drinking, 2 = no, not interested, 3 = No, but he/she tried it, 4 = I think he/she does, 5 = I found out he/she does, 6 = Yes, he/she drinks regularly, 9 = I don't know. The answers options 1 and 2 were recoded into 0 = Never using, 3 was recoded into 1 = Not using but has experience, 4 into 2 = I think he/she drinks, 5 into 3 = I found out he/she drinks, and 9 was coded as missing (no valid answers using 6). Pediatricians reported whether adolescents used alcohol (yes or no). If they answered yes, this was followed with a question about frequency: 1 = a glass of beer or wine not more than once a month, 2 = a larger dose not more than once a month, 3 = a glass of beer or wine more than once a month, 4 = a larger dose more than once a month. Responses 1 + 2 were recoded into one category, resulting in four responses options, ranging from Does not drink (0) to More than one drink more than once a month (3).

#### 3.2.2 Exposures

*Family SES.* Taken at baseline. The family SES was operationalized as a latent factor using the following three indices from study baseline: maternal educational level, paternal educational level, and paternal occupational status. Regarding education level, mothers and fathers reported their highest attained education, coded as 0 = less than high school, 1 = vocational school, 2 = high school degree, 3 = some college, 4 = college degree, and 5 = PhD degree. The occupational status of fathers was coded using the International Standard Classification of Occupations (ISCO), which was then recoded into a continuous International Socio-Economic Index of Occupational Status (ISEI), with higher values indicating higher status (Ganzeboom et al., 1992).

*Neighborhood advantage.* The measure of neighborhood advantage used indices from the 2001 Czech census when the children were around 9 years of age, the closest available census data to our outcome variable (at age 15). Neighborhood advantage indices were aggregated at the level of Basic Settlement Unit (BSU), defined by the Czech Statistical Office as the smallest standardized territorial unit, characterized as part of a municipal area with clearly defined spatial characteristics. There was a total of *n* = 173 BSU clusters in the current sample. Neighborhood advantage was operationalized as a latent factor with the following indices: percentage of residents with a college degree, percentage of unemployed adults in economically active age (reverse), and percentage of households reporting ownership of a telephone, car, and access to Internet. Although the latter index might be less relevant today, it represented a meaningful marker of socioeconomic status during the time of 2001 Census, when home access to Internet was relatively uncommon and much more expensive.

*Neighborhood disorder.* Reported at age 15 of the child, neighborhood disorder was self-rated by mothers by reporting on their neighborhood using seven items, asking about *“How big of a problem for your living are the following”* with the following options: *noise from the street*, *noisy youth*, *street clutter*, *dog dirt on the sidewalks*, *vandalism*, *theft*, *robbery*. Each option was rated on a 3-point scale, where 1 = serious problem, 2 = small problem, 3 = no problem. We dichotomized the response options where options 2 and 3 were merged and recoded into 0, making this measure reflect only serious problems with each category. These seven categorical items were then used as indicators of a latent factor.

#### 3.2.3 Mediators

*Peer* *alcohol use.* This was self-reported by adolescent at age 15 by answering the question: “I have friends who … get drunk often” with the options: yes (1) and no (0).

*Maternal alcohol use*. Reported at age 15 of the adolescent. Mothers reported the count of drinks they had in the previous week in the following categories: beers, cocktails, liquors, wine. The number of drinks across categories were summed and this was then categorized into the following: 0 drinks = 1, 1 drink = 2, 2 to 6 drinks = 3, 7:14 drinks = 4, 15 drinks and more = 5.

*Paternal alcohol use.* Reported at age 15 of the adolescent. Mothers reported on their partners’ alcohol use by responding to the item *Which of the following statements about alcohol best describes your partner?* using these response options: 1 = he never drinks alcohol, 2 = he drinks alcohol less than once a week, 3 = he drinks occasionally, at least once a week, 4 = one or two drinks almost every day, 5 = 3 to 9 drinks every day, 6 = 10 or more drinks every day. Responses 5 + 6 were merged due to low count for category 6.

*Maternal monitoring.* Reported at age 15 of the adolescent by mothers by answering the question *Do you know where your teenager is when he/she is outside* using the following responses: 1 = I always know, 2 = I usually know, 3 = I seldom know, 4 = I never know. This was recoded so that higher values indicated more maternal knowledge.

#### 3.2.4 Confounders

*Sex*. Reported at the study baseline. Mother-reported sex of the adolescents was coded as 0 = male, 1 = female.

*Family structure*. Reported at the study baseline. Coded as 1 = two adults living together, whereas other family structures were coded as 0.

## 4 Plan of analysis

First, descriptive statistics of the study variables were computed. Then, structural equation modeling (SEM) was used to examine the links between SES, neighborhood advantage, and neighborhood disorder. The main outcome (adolescent alcohol use at age 15) was modeled as a latent construct with the three reporters’ responses as its indicators. Given its low ICC (<1.5%), we decided to model the clustering of individuals in BSUs in all subsequent inferential analyses by adjusting the standard errors for clustering using Taylor series linearization but not partitioning the outcome variance directly through multilevel modeling. SES was conceptualized as a latent construct composed of maternal education, paternal education, and paternal occupational status (ISEI). Neighborhood advantage was modeled as a latent construct indicated by the proportion of residents in the BSU with a college degree, unemployment rate of the BSU, and the proportion of households in the BSU owning a phone, car, and Internet connection. Neighborhood disorder was modeled as a latent construct with the 7 dichotomized items as indicators.

We tested our hypotheses by incrementally expanding the structural model. First, we regressed the outcome separately on main exposure variables (SES, neighborhood advantage, neighborhood disorder). Then, we regressed the neighborhood advantage and neighborhood disorder on SES and alcohol outcome on all three to see if the hypothesized effect of SES on adolescent alcohol use is explained by the neighborhood effects. After this, we added confounders (sex, family structure) to the model.

In the final step, we estimated the full model with proximal mediators included, predicting alcohol use and regressed on family SES, neighborhood advantage, and neighborhood disorder, with all endogenous variables in the model also regressed on covariates. We estimated several indirect effects in a complex mediation model, including both serial and parallel mediation (SES→neighborhood advantage/disorder→proximal mediators→adolescent alcohol use. To test the significance of indirect effects, a bootstrapping procedure with 5000 resamples and bias-corrected confidence intervals was estimated. All models were run in Mplus 8.10 using weighted least squares mean and variance adjusted (WLSMV) estimator, which is appropriate for modeling categorical outcomes and accounts for non-normality in the data. Missing data were handled via pairwise present-data estimation, consistent under the assumption that missingness depends only on variables included in the model (the MAR condition; Asparouhov & Muthén, 2010). Exogenous covariates with missing data (sex, family structure) were modeled with estimated variances/covariances rather than as fixed predictors, extending this treatment to them (Muthén & Muthén, 1998-2017).

## 5 Results

Descriptive statistics of the study sample are provided in Table 1. At age 15, about 16% of adolescents have never tried alcohol based on their reports, with about 70% having used it more than once or often. Mother's report on adolescent alcohol use is much lower than adolescents' own, reporting 32.9% of adolescents never using alcohol by the age of 15. Pediatrician report seems to be somewhat in between these estimates but again underestimated compared to adolescent report, with half of the valid sample reported as non-drinkers.

**Table 1:** Descriptive statistics of study variables.

		*n*	%	Valid %
Sex				
	Female	758	49.6%	49.6%
	Male	770	50.4%	50.4%
	Missing	0	0.0%	
Family structure				
	Single-parent family	172	11.3%	12.1%
	Two-parent family	1244	81.4%	87.8%
	Missing	112	7.3%	
Maternal education				
	Less than high school	39	2.6%	3.1%
	Vocational school	281	18.4%	22.5%
	High school	539	35.3%	43.2%
	Some college	78	5.1%	6.2%
	College degree	284	18.6%	22.8%
	Doctorate	27	1.8%	2.2%
	Missing	280	18.3%	
Paternal education				
	Less than high school	27	1.8%	2.2%
	Vocational school	364	23.8%	30.2%
	High school	368	24.1%	30.5%
	Some college	37	2.4%	3.1%
	College degree	361	23.6%	30.0%
	Doctorate	48	3.1%	4.0%
	Missing	323	21.1%	
Adolescent report of alcohol use				
	Never	227	14.9%	16.3%
	I used it only once	158	10.3%	11.3%
	I used it more than once	903	59.1%	64.7%
	I use it often	108	7.1%	7.7%
	Missing	132	8.6%	
Mother's report of adolescent's alcohol use				
	Never used	486	31.8%	32.9%
	Not using but has experience	905	59.2%	61.3%
	I think he/she drinks	59	3.9%	4.0%
	I found out he/she drinks	26	1.7%	1.8%
	Missing	52	3.4%	
Pediatrician report of ad. alcohol use				
	Does not drink	406	26.6%	56.3%
	One drink or more not more than once a month	267	17.5%	37.0%
	One drink more than once a month	25	1.6%	3.5%
	More drinks more than once a month	23	1.5%	3.2%
	Missing	406	26.6%	
Neighborhood disorder	Noise from the street	1513	10.5%	10.6%
	Noisy youth	1512	5.5%	5.6%
	Street clutter	1513	17.0%	17.2%
	Dog dirt on the sidewalks	1512	37.6%	38.0%
	Vandalism	1513	27.3%	27.6%
	Theft	1512	21.1%	21.3%
	Robbery	1511	14.2%	14.4%
	Missing	15	<0.1%	
Mother's alcohol use				
	no drinks	448	29.3%	29.5%
	1 drink a week	203	13.3%	13.4%
	2 to 6 drinks a week	679	44.4%	44.8%
	7 to 14 drinks a week	164	10.7%	10.8%
	more than 15 drinks a week	23	1.5%	1.5%
	Missing	11	0.7%	
Father's alcohol use				
	Never drinks	89	5.8%	6.8%
	Drinks less than once a week	440	28.8%	33.6%
	Drinks sometimes, at least once a week	478	31.3%	36.5%
	Drinks one or two drinks almost every day	259	17.0%	19.8%
	Drinks 3 or more drinks every day	42	2.7%	3.2%
	Missing	220	14.4%	
Maternal monitoring				
	I never know	8	0.5%	0.5%
	I seldom know	88	5.8%	5.9%
	I usually know	963	63.0%	64.8%
	I always know	426	27.9%	28.7%
	Missing	43	2.8%	

Peers using alcohol	No	916	59.9%	68.8%
	Yes	416	27.2%	31.2%
	Missing	196	12.8%	
		n	Mean (SD)	Min/max

Paternal ISEI		1050	49.49 (22.14)	13.3/89.0
Percentage of college-educated adults		1524	17.9% (5.91)	2.2%/40.1%
Percentage of unemployed		1524	4.5% (1.92)	0.9%/18.4%
Percentage of household owning car, telephone, and access to Internet		1524	6.6% (2.13)	0.0%/13.89%

Regarding the exposure variables, about 25% of mothers and 34% of fathers had college degree, while about 25% of mothers and 32% of fathers with less than high school education. The father's mean ISEI was 49.5, with a minimum of 13.3 (livestock farmer) and maximum of 89.0 (judge). The most commonly endorsed indicator of neighborhood disorder was dog dirt on sidewalks (38.0%) and vandalism (27.6%). Regarding the objective neighborhood data, the average BSU rate of college-educated adults was 17.9%, average unemployment rate was 4.5%, and only about 6.6% of households on average reported owning car, telephone, and Internet access.

Turning to mediators, about 29% of mothers reported that they never drink by child's age of 15, while only 5.8% of fathers were non-drinkers. More than 12% of mothers drank one or more drinks each day, while more than 22% of fathers drank one or more drinks per day. Regarding maternal monitoring, more than 90% of mothers reported that they usually or always know where their children were (with less than 1% never knowing). A total of 31.2% of adolescents reported that they had friends who get drunk often around the age of 15.

In the next step, we tested the direct effects of SES, neighborhood disorder and neighborhood advantage on latent alcohol use in structural models. The results are shown in Table 2.

**Table 2:** Bivariate and multivariate models predicting adolescent alcohol use.

	Beta	*SE*	*p*	*R*^*2*^
Bivariate				
Family SES	0.03	0.04	0.540	0.001
Neighborhood advantage	−0.03	0.03	0.405	0.001
Neighborhood disorder	−0.16	0.04	<0.001	0.027

Multivariate				
Family SES	0.02	0.04	0.702	
Neighborhood advantage	−0.07	0.04	0.067	
Neighborhood disorder	−0.18	0.04	<0.001	
				0.031
Multivariate adjusted				
Family SES	0.02	0.05	0.691	
Neighborhood advantage	−0.06	0.04	0.088	
Neighborhood disorder	−0.17	0.04	<0.001	
Female	0.07	0.03	0.028	
Two-parent family	0.01	0.03	0.718	
				0.036

*Note.* Multivariate adjusted structural model included effects of sex and family structure on the outcome as well as on neighborhood advantage and neighborhood disorder.

All latent factors (neighborhood advantage, neighborhood disorder, adolescent alcohol use) showed adequate factor loadings (all λ > |0.64|). Family SES was not associated with adolescent alcohol use, β = 0.03, *p* = .540. There was no significant association between neighborhood advantage and adolescent alcohol use (β = −0.03, *p* = .405). On the other hand, neighborhood disorder was negatively associated with alcohol use (β = −0.16, *p* < .001) showing that neighborhoods with more disorder had lower levels of adolescent alcohol use.

Then, neighborhood advantage and neighborhood disorder were regressed on family SES and included in the same multivariate model. SES was not significantly associated with alcohol use (β = 0.02, *p* = .702) while it was positively associated with neighborhood advantage (β = 0.26, *p* < .001) and negatively associated with neighborhood disorder β = −0.16, *p* < .001. Neighborhood disorder remained a negative predictor of alcohol use, (β = −0.18, *p* < .001) while neighborhood advantage was not statistically significantly associated with alcohol use. Neighborhood advantage and neighborhood disorder were significantly negatively correlated, *r* = −0.18, *p* < .001. In the third step, we regressed adolescent alcohol use and neighborhood characteristics on covariates (sex, family structure). The previous findings from the unadjusted multivariate model remained practically unchanged. Higher levels of alcohol use at age 15 were reported for girls, β = 0.07, *p* = .028.

**Fig. 1:**
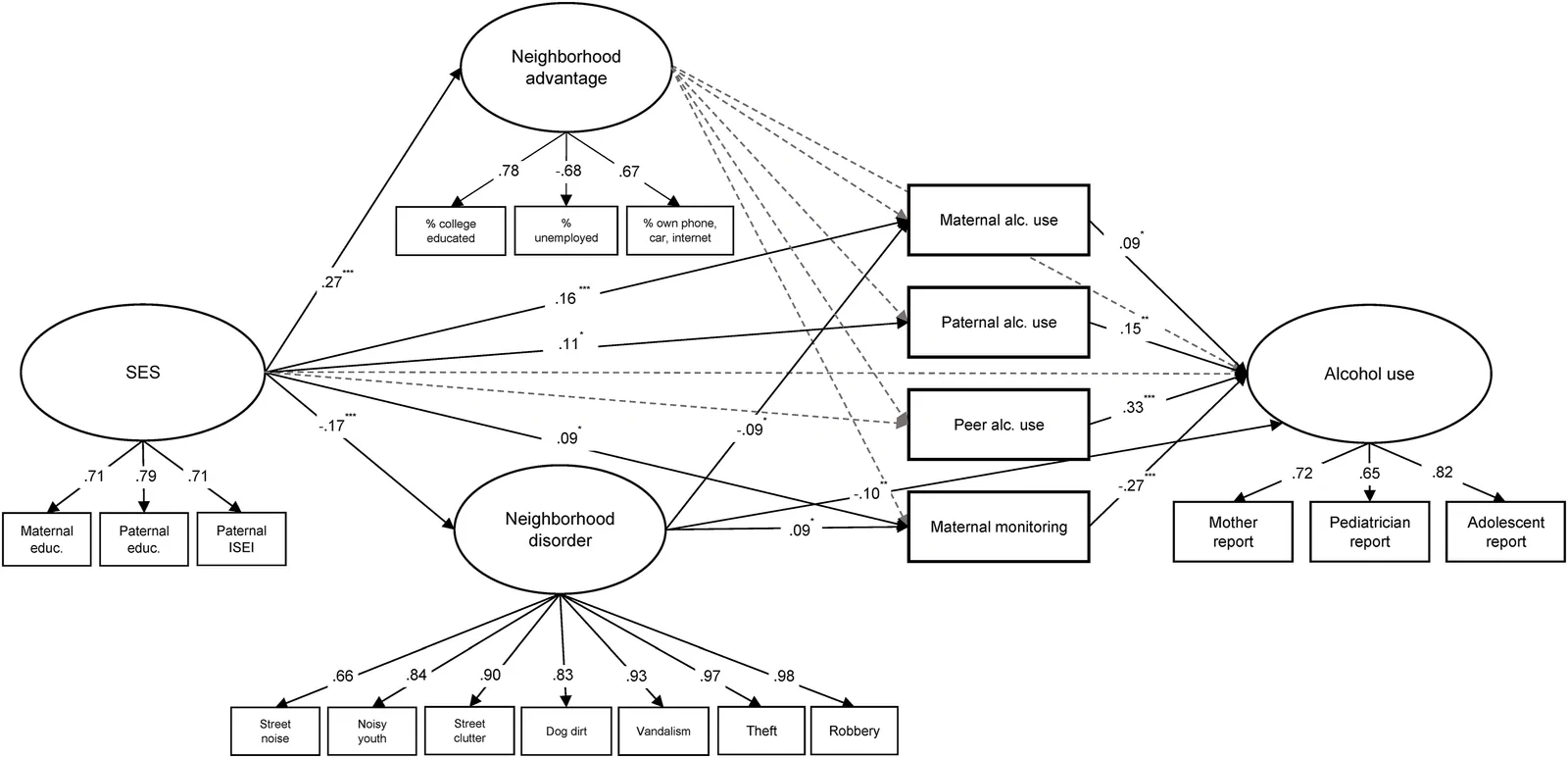
Results from the full structural model. *Note*. Standardized estimates. The correlations among mediators and neighborhood characteristics not shown but estimated. Dashed lines are paths not statistically significant at p < .05. All factor loadings significant at *p* < .001. *p < .05, **p < .01, ***p < .001 Neighborhood disadvantage, neighborhood disorder, alcohol use and the mediators were also regressed on covariates (sex, family structure, maternal education) – not shown here.

Next, we estimated the full structural model with both exposure variables and all mediators assessed at the same time. The standardized results for the statistically significant paths are shown in Fig. 1. Full results including all estimates from the model are part of Appendix A. The fit of the model was adequate, χ2 (170) = 426.90, CFI = 0.98, RMSEA = 0.03 [0.03, 0.04], TLI = 0.98, SRMR = 0.051. In the full model, a direct effect of neighborhood disorder on alcohol use remained, β = −0.10, *p* = .007 while no statistically significant direct effects were observed for family SES and neighborhood advantage. All proposed mediators of neighborhood effects were found to be associated with adolescent alcohol use: peer drinking (β = 0.33, *p* < .001), maternal drinking (β = 0.10, *p* = .012), and paternal drinking (β = 0.15, *p* = .002) predicted higher alcohol use while maternal monitoring (β = −0.27, *p* < .001) was negatively associated with adolescent drinking.

Levels of maternal monitoring were found to be higher among higher SES families (β = 0.09, *p* = .039) and, conversely, in neighborhoods with higher disorder (β = 0.09, *p* = .017). Having friends who get drunk often was not found to be affected by the exposures of interest. Maternal drinking quantity was lower in neighborhoods with higher disorder (β = −0.09, *p* = .022) and higher in families with higher SES (β = 0.16, *p* < .001). Paternal drinking was also positively associated with SES (β = 0.11, *p* = .038).

Finally, we turned to testing the indirect effects. First, SES was found to have a positive indirect effect through maternal drinking (β = 0.014 95% BcCI [0.003, 0.034]) as well as through paternal drinking (β = 0.016 [0.002, 0.043]), and a negative effect through maternal monitoring, β = −0.024 [−0.051, −0.003]. Furthermore, SES was associated with higher alcohol drinking through neighborhood disorder (β = 0.017 [0.004, 0.036]), and this indirect effect was also stat. significant for serial mediation through neighborhood disorder and maternal monitoring, β = 0.004 [0.001, 0.011]. A significant negative indirect effect of neighborhood disorder through maternal drinking (β = −0.008 [−0.023, −0.001]) was found. In addition, an indirect effect through maternal monitoring was statistically significant in the full model, β = −0.025 [−0.051, −0.005]. No effect through neighborhood advantage or from neighborhood advantage was statistically significant.

The full model explained 31.1% of variance in adolescent alcohol use.

### 5.1 Sensitivity analysis

Given the likely possibility that mothers and pediatricians underreported adolescent alcohol use, the self-report measure could be the most valid single indicator of the outcome. We therefore re-estimated the full model using the adolescent self-report alone as the outcome measure. The results were largely consistent with the main analysis: neighborhood disorder significantly predicted adolescent alcohol use (β = −0.08, *p* = .017), as did peer alcohol use (β = 0.37, *p* < .001), maternal monitoring (β = −0.17, *p* < .001), and paternal drinking (β = 0.12, *p* = .007). Two differences emerged relative to the main findings. First, SES was directly and significantly associated with alcohol use (β = 0.11, *p* < .001), a relationship that was not significant in the primary analysis. Second, maternal alcohol use was no longer a significant predictor (β = 0.04, *p* = .281), and the indirect effects operating through maternal and paternal drinking did not reach statistical significance. All other indirect effects reported in the main analysis remained statistically significant. Taken together, these findings suggest that the core conclusions of the study are robust to the choice of outcome measure, with the noted exception of the pathways involving parental drinking.

## 6 Discussion

The present study sought to evaluate the joint effect of family SES, neighborhood advantage, and neighborhood disorder on adolescent alcohol use, proposing multiple proximal mediators to explain these associations. Results from structural equation modeling showed that higher family SES was indirectly associated with higher adolescent alcohol use through higher levels of maternal as well as paternal alcohol use and maternal monitoring, while it was negatively associated with neighborhood disorder, which was in turn found to be associated with lower levels of alcohol use. No direct or indirect effects were found for neighborhood advantage. Peer alcohol use was an independent positive predictor of higher levels of adolescent alcohol use.

Family SES was related to higher levels of parental drinking, both for mothers and fathers. Parental drinking in turn predicted higher levels of alcohol use among their adolescent children, and the indirect effect of SES was significant and positive. This finding confirms results of many previous studies which found that individuals with higher SES engage in more frequent and heavier drinking (Barr, 2018; Collins, 2016; Demidenko et al., 2024) and that parental alcohol use predicts alcohol use of their children (Song et al., 2012).

Family SES was positively associated with neighborhood advantage, showing that the administrative indices used to reflect neighborhood advantage were relevant. Furthermore, neighborhood advantage was negatively associated with neighborhood disorder, confirming previous literature showing that physical disorder and higher levels of perceived crime are found in less advantaged neighborhoods, even though neighborhood disadvantage was based on subjective perceptions of mothers. However, neighborhood advantage was not associated with any of the proposed mediators or directly with adolescent alcohol use, in contrast to some previous studies, which found neighborhood advantage to be positively associated with more alcohol drinking (Lo et al., 2006; Slutske et al., 2016), but in line with other studies that did not find associations between these two variables.

The results also showed that subjective experience of neighborhood disorder was a stronger predictor of adolescent alcohol use than the objective measure of neighborhood advantage. Although neighborhood disorder has been much less studied compared to disadvantage, there are studies that found it to be associated with higher problem behaviors (Harlow et al., 2025; Jackson et al., 2016). This suggests that subjective perception, although with more potential for bias, might nevertheless be a more relevant proxy for neighborhood characteristics than official administrative data as it describes how actual residents perceive their surroundings. The lack of finding for objectively measured neighborhood advantage might be due to the fact that administratively-defined BSUs in this study would not completely overlap with what one considers their neighborhood, a common issue observed in other related studies (Basta et al., 2010).

However, the current results showed that higher perceived levels of neighborhood disorder were associated with lower levels of drinking among adolescents. This result is in contrast to tenets of Social disorganization theory but in line with multiple other studies that also found this negative association (Demidenko et al., 2024; Slutske et al., 2016).

Further, it aligns with the negative link between family SES and neighborhood disorganization in the current study as families with higher SES had higher rates of alcohol drinking and were also less likely to live in disorganized neighborhoods. One possible explanation is that disordered neighborhoods offer fewer opportunities for social activities and gatherings in which alcohol is typically present, relative to more orderly neighborhoods (Pedersen et al., 2015; Zeng et al., 2026). Given the relatively high prevalence of adolescent drinking in this sample, which likely reflects broad social acceptance of alcohol use, reduced exposure to such occasions in disordered neighborhoods may make early experimentation with alcohol comparatively less normative for adolescents growing up there (Snedker et al., 2009).

Interestingly, we found a paradoxical effect involving family SES, maternal monitoring, and neighborhood disorder: higher SES was associated with higher levels of monitoring, and neighborhood disorder was also associated with higher monitoring, even though family SES was linked to lower neighborhood disorder. This is an example of a suppression effect, where the inclusion of neighborhood disorder actually increased the association between SES and maternal monitoring. This suggests the existence of two pathways to monitoring. The first is a direct, resource-driven pathway where higher SES parents engage in more monitoring, having the resources (time, knowledge) to do so, in line with many previous studies (Farley & Kim-Spoon, 2017). The second is an indirect, risk-driven pathway where parents in more disordered neighborhoods may increase monitoring as a protective response against the perceived risks (Chuang et al., 2005; Sullivan et al., 2004).

Neither objective nor subjective characteristics of neighborhoods were related to likelihood of affiliating with drinking peers, itself a strong predictor of drinking in this study, as well as in many other studies (Obradors-Rial et al., 2020). Given the relative ubiquity of underage alcohol drinking in the Czech Republic (Czech Statistical Office, 2020; Hodačová et al., 2017), it is possible that alcohol-drinking peers do not specifically concentrate in either advantaged or disordered neighborhoods in this sample.

The implications of this study findings suggest that individual lived and perceived experience is just if not more important than objectively assessed neighborhood qualities, as far as it relates to problem behaviors, highlighting the importance of incorporating lived experiences into neighborhood research. This is because what constitutes neighborhood is itself a subjective quality and administrative boundaries might only approximate this. For this reason, it is important that individual neighborhood assessments complement objective neighborhood characteristics in future studies.

Family context, including parental alcohol use and monitoring, emerged as important proximal mediators of the neighborhood disorder. Alcohol use had very little variance explained at the neighborhood level, suggesting that individual and family-level factors may be more predictive of this behavior than more distal contexts. Although the operationalization of parental monitoring in this study was rather crude, it remained a robust predictor of adolescent alcohol use in the full model with multiple factors included. In this way, monitoring remains one of the most important protective factors against risky behaviors in adolescence. However, our own findings show that higher SES was directly associated with greater parental monitoring, suggesting that parents’ capacity to supervise may itself depend on the resources (e.g., time, flexibility, knowledge) that socioeconomic advantage affords. Preventive interventions that strengthen parental involvement and supervision should therefore be paired with efforts to address the upstream social and economic constraints that limit this capacity, particularly for families facing socioeconomic disadvantage. Importantly, our findings show that parental alcohol use mediated the link between higher family SES and adolescent alcohol use, suggesting that substance use prevention efforts cannot rely solely on targeting socioeconomically disadvantaged populations. Higher SES may confer greater access to alcohol and be associated with more permissive norms around its use. Interventions should target the normative acceptability of alcohol use in more affluent households and emphasize the role of parental behavior—not only in terms of monitoring but also in their own substance use. Public health messaging should emphasize the role-modeling aspect of parental alcohol use and challenge the common belief that supplying children with alcohol in home environment teaches them responsible drinking (Ksinan et al., 2023).

### 6.1 Strengths and limitations

A major strength of this study is its complex and integrative design. It examines the longitudinal effect of early-life family SES on adolescent alcohol use 15 years later and simultaneously models the effects of objective and subjective neighborhood characteristics, multiple mediators, and multiple reporters of the key outcome. Our proposed pathways showed links between family SES, neighborhood characteristics, proximal risk factors for drinking, and alcohol use in adolescence. The inclusion of multiple variables enabled us to disentangle the many interrelated factors hypothesized to affect alcohol use in adolescence, such as parental or peer alcohol use. Including these factors as intermediary variables rather than controls provided evidence towards causal pathways and improves the validity of the findings.

Another important advantage of this study is its Central European setting, as vast majority of studies on neighborhood effects and problem behaviors have been carried out in Western countries (especially United States). Including a Czech sample enhances the cross-cultural validity of neighborhood theories and contributes valuable evidence on adolescent alcohol use in a high-consumption, post-socialist setting with relatively low levels of income inequality and high alcohol availability.

Nevertheless, several limitations should be acknowledged. A key concern in mediation analyses where mediators and the outcome share the same measurement occasion is that temporal ordering cannot be guaranteed. However, this limitation applies unevenly across the variables in our model. For neighborhood disorder, reverse causality is substantively implausible: it is difficult to argue that adolescent alcohol use causes the physical and social deterioration of a neighborhood. Focusing on an earlier timepoint (age 11) was possible, yet this choice would introduce a different problem, namely that neighborhood characteristics may have changed meaningfully in the four years between assessment and outcome, potentially misrepresenting the neighborhood context adolescents actually experienced at age 15. For the proximal mediators (such as peer drinking), the concurrent assessment is a more genuine limitation, as bidirectional influences between peer behavior and adolescent alcohol use are plausible. Unfortunately, these variables were not assessed prior to age 15, making a temporally lagged approach infeasible within the available data.

Our decision to focus on the age 15 timepoint reflects a necessary trade-off between methodological rigor and empirical feasibility. At this age, alcohol use was prevalent enough to allow meaningful analysis of the outcome while the sample size remained sufficiently large to support the complexity of the structural model used. In contrast, the next available measurement wave at age 18 suffers from substantial attrition (available N ∼ 700), which would compromise statistical power and potentially introduce selection bias. Therefore, while the concurrent assessment of variables at age 15 poses a limitation, we believe it represents a justifiable and pragmatic choice within the constraints of the data. Nevertheless, it remains important to examine how the influence of neighborhood context unfolds over time, particularly in relation to early-life neighborhood conditions and their impact on outcomes in adulthood.

Sample attrition was a limitation of this study, as the analytic sample comprised approximately a third of the original baseline sample. The participants who remained were of higher socioeconomic status compared to the baseline sample, restricting the range of SES in the analytic sample. As a result, the reported associations involving SES may be conservative estimates of the true effect in the general population.

The absence of some important mediators at the neighborhood and individual level is a limitation of the study. Specifically, our data did not include information on the availability of alcohol, either objectively measured (such as alcohol retail outlet density; Bryden et al., 2012; Jackson et al., 2014) or perceived ease of obtaining alcohol (especially in the underage population; Kuntsche et al., 2008; Treno et al., 2008). These are important intervening factors that have been found to be positively associated with higher levels of alcohol use and should be included in future studies to provide full picture of risk factors for adolescent alcohol use.

Finally, it should also be noted that the pathways identified here represent average associations across the whole sample; these effects may not operate uniformly across different social groups, and future research should test for such moderation directly.

## 7 Conclusions

This study examined how family socioeconomic status (SES), neighborhood characteristics, and individual and family-level factors jointly influence adolescent alcohol use in a large Central European cohort. Our findings underscore the complexity of these relationships. Family SES was indirectly and positively associated with adolescent alcohol use through parental drinking, maternal monitoring, and neighborhood disorder. While objective neighborhood advantage did not predict alcohol use, subjective neighborhood disorder was associated with lower levels of adolescent drinking. Neighborhood disorder was also positively associated with higher maternal monitoring, which was predictive of lower levels of alcohol use. Furthermore, association with drinking peers was an independent predictor of higher adolescent alcohol use. Together, these results suggest that effective strategies for preventing adolescent alcohol use should move beyond targeting structurally disadvantaged groups and also address cultural norms and behaviors within higher SES families.

## CRediT authorship contribution statement

**Albert J. Ksinan:** Conceptualization, Formal analysis, Funding acquisition, Methodology, Validation, Visualization, Writing – original draft. **Daniel Szabó:** Conceptualization, Methodology, Writing – original draft. **Lenka Andrýsková:** Funding acquisition, Methodology, Project administration, Writing – original draft. **Martin Bobak:** Conceptualization, Formal analysis, Investigation, Methodology, Supervision, Writing – original draft. **Hynek Pikhart:** Conceptualization, Formal analysis, Methodology, Supervision, Writing – original draft.

## Ethical statement

The secondary use of all ELSPAC study data was approved by the (C)ELSPAC Ethics Committee at Masaryk University in Brno, Czech Republic (Ref. No. ELSPAC/EK/1/2014, date 09/17/2014).

The privacy rights of human subjects have been observed and Informed consent was obtained from all participants in the study.

## Conflict of interest

The authors declare no conflict of interest.

## Data Availability

Data will be made available on request.
